# Supplementary figures and images for: Using networks to analyze and visualize the distribution of overlapping genes in virus genomes
Source: PLoS Pathog. 2022 Feb 24;18(2):e1010331. doi: 10.1371/journal.ppat.1010331 (PMC8903798; doi:10.1371/journal.ppat.1010331)

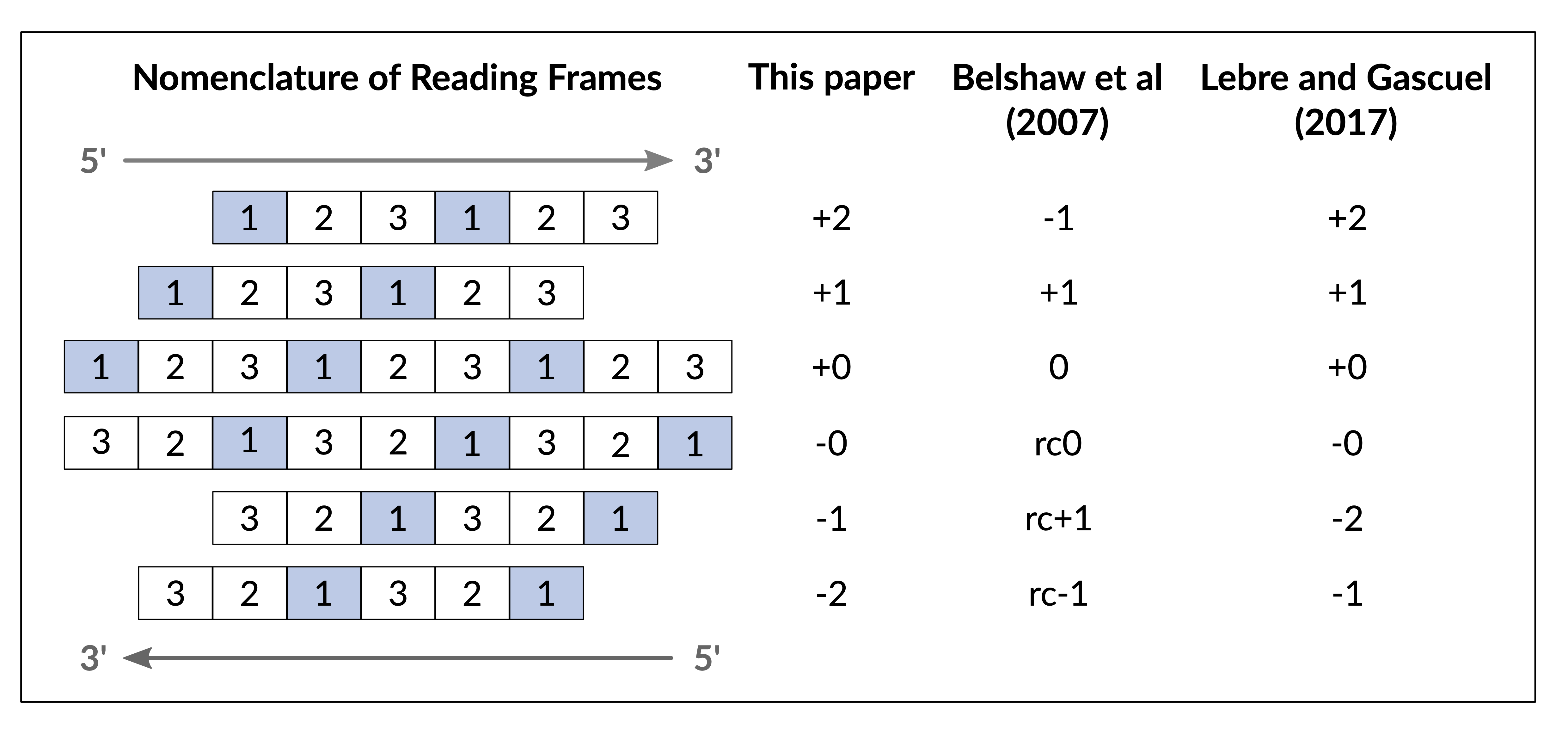

Supplement: S1 Fig — (TIFF) [file ppat.1010331.s001.tiff]

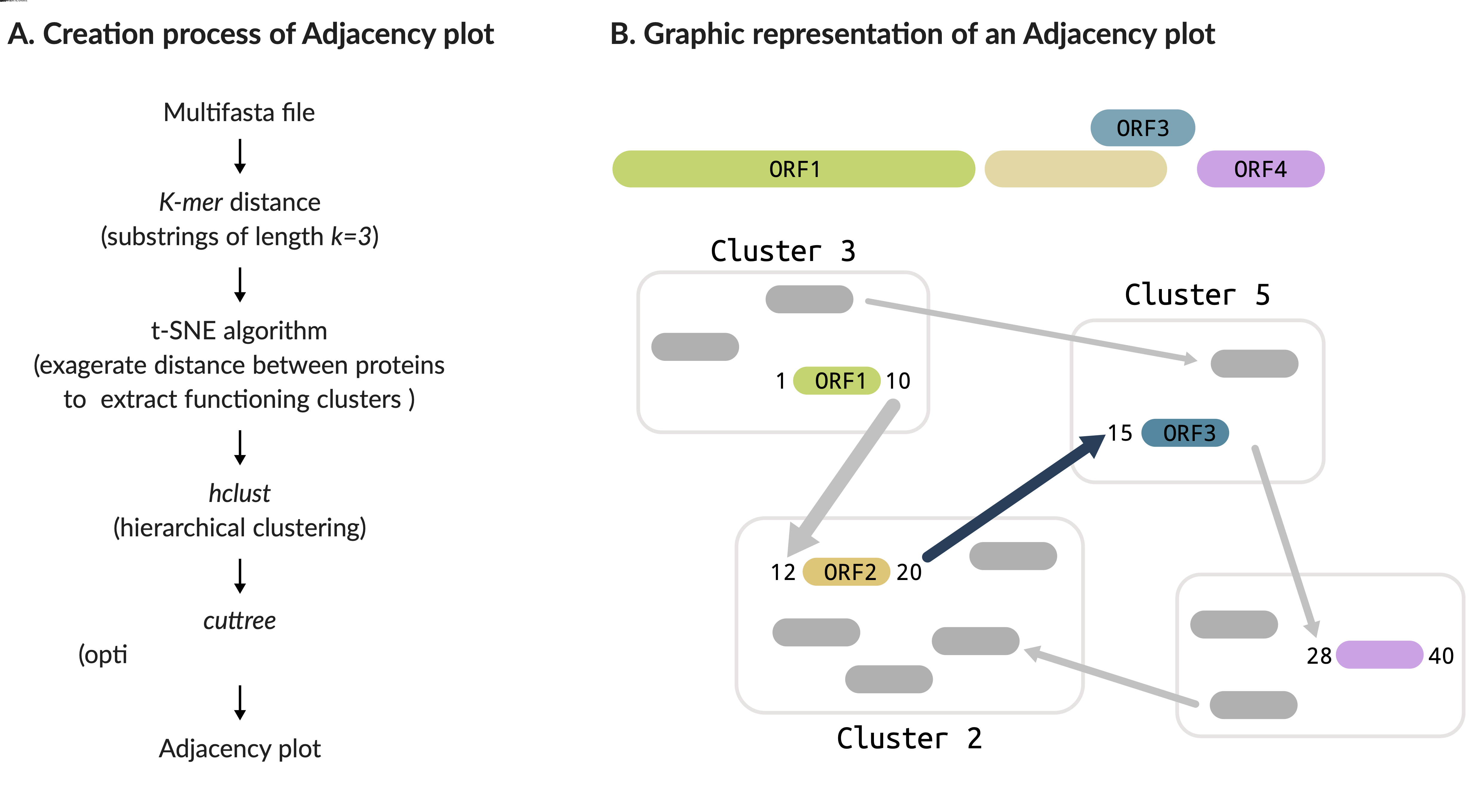

Supplement: S3 Fig — A. Steps used to generate the input file for the adjacency plot. First, we downloaded a multifasta file containing the protein sequences of the reference genomes for each species in the virus family. Then, we used a Python script to calculate the k-mer distance between proteins followed by an R script to designate each protein to a cluster according to homology. Finally, we used a Python script to generate dot files using Graphviz. B. Adjacency plot interpretation. Each one of the proteins that constitute a genome is assigned to a different cluster with homologous proteins from other species. From each cluster, we draw arrows in gray that represent adjacent proteins and arrows in blue that represent overlapping proteins. The width of the arrow is proportional to the number of proteins related between the two clusters. One cluster can have entries adjacent to proteins in different clusters. In this example, cluster 3 has proteins adjacent to proteins in cluster 2 and cluster 5. (TIFF) [file ppat.1010331.s003.tiff]

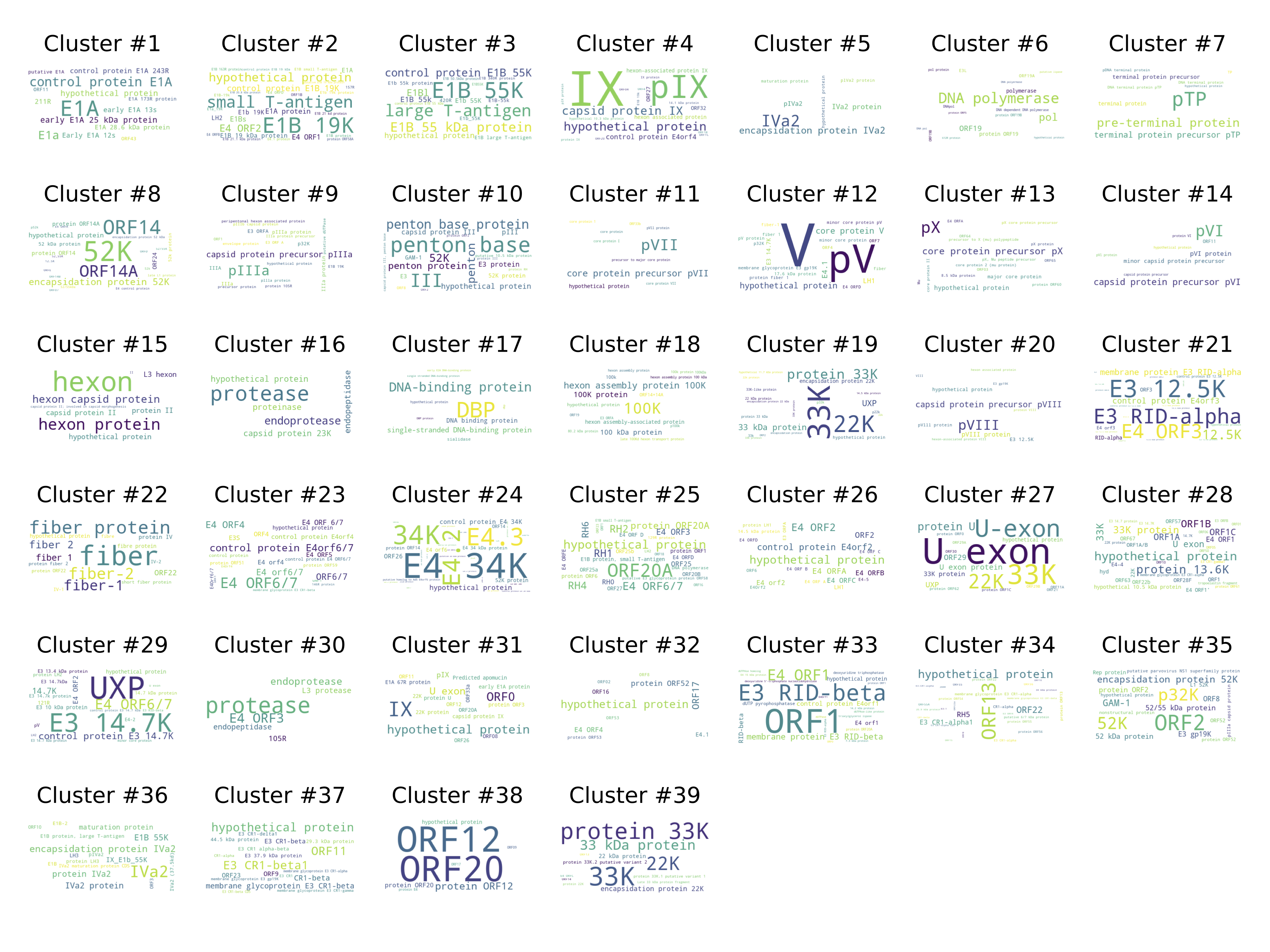

Supplement: S4 Fig — The size of each word (gene annotation) is scaled in proportion to its relative frequency in association with ORFs in the respective clusters. (TIFF) [file ppat.1010331.s004.tiff]

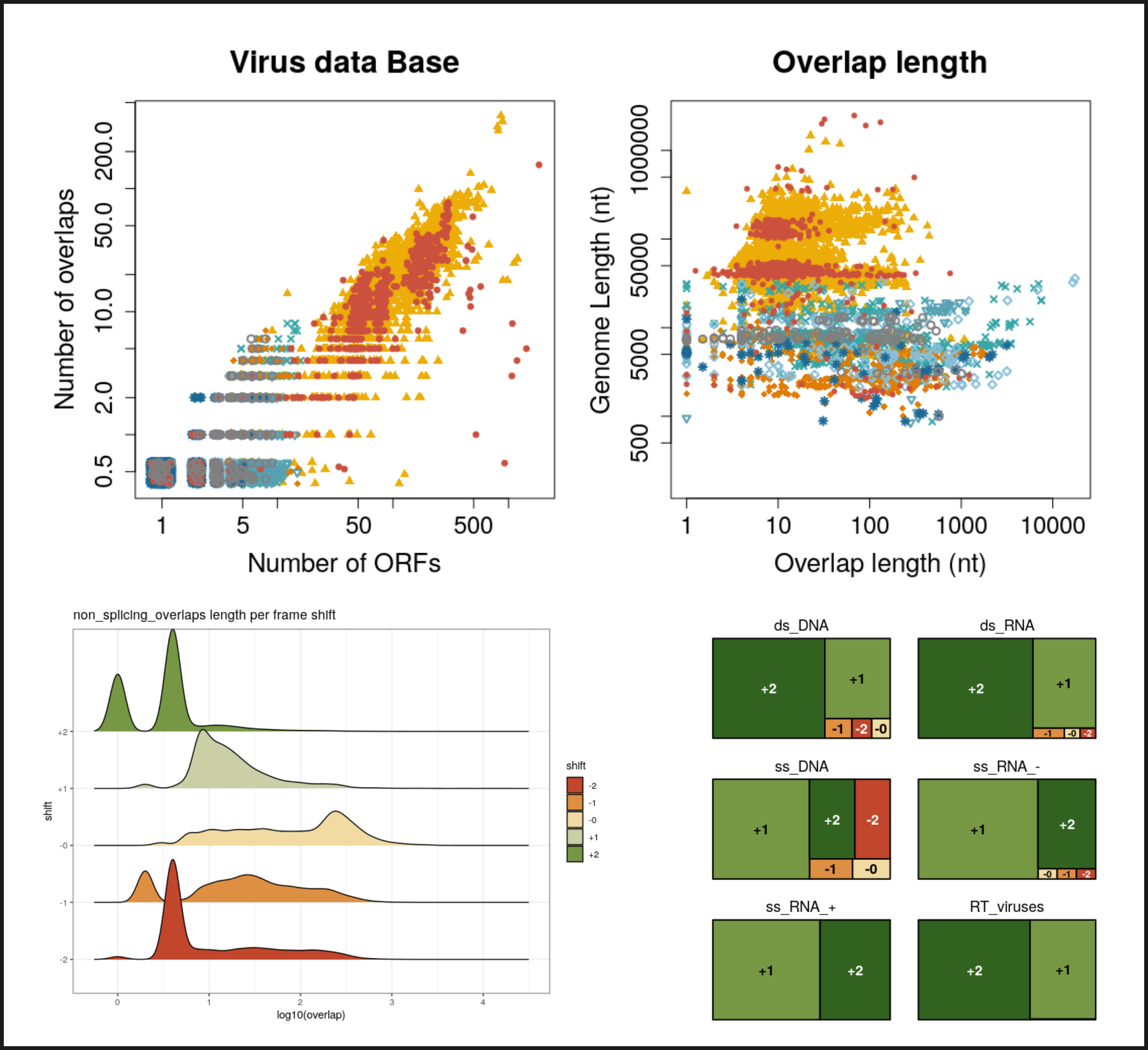

Supplement: S5 Fig — (TIFF) [file ppat.1010331.s005.tiff]

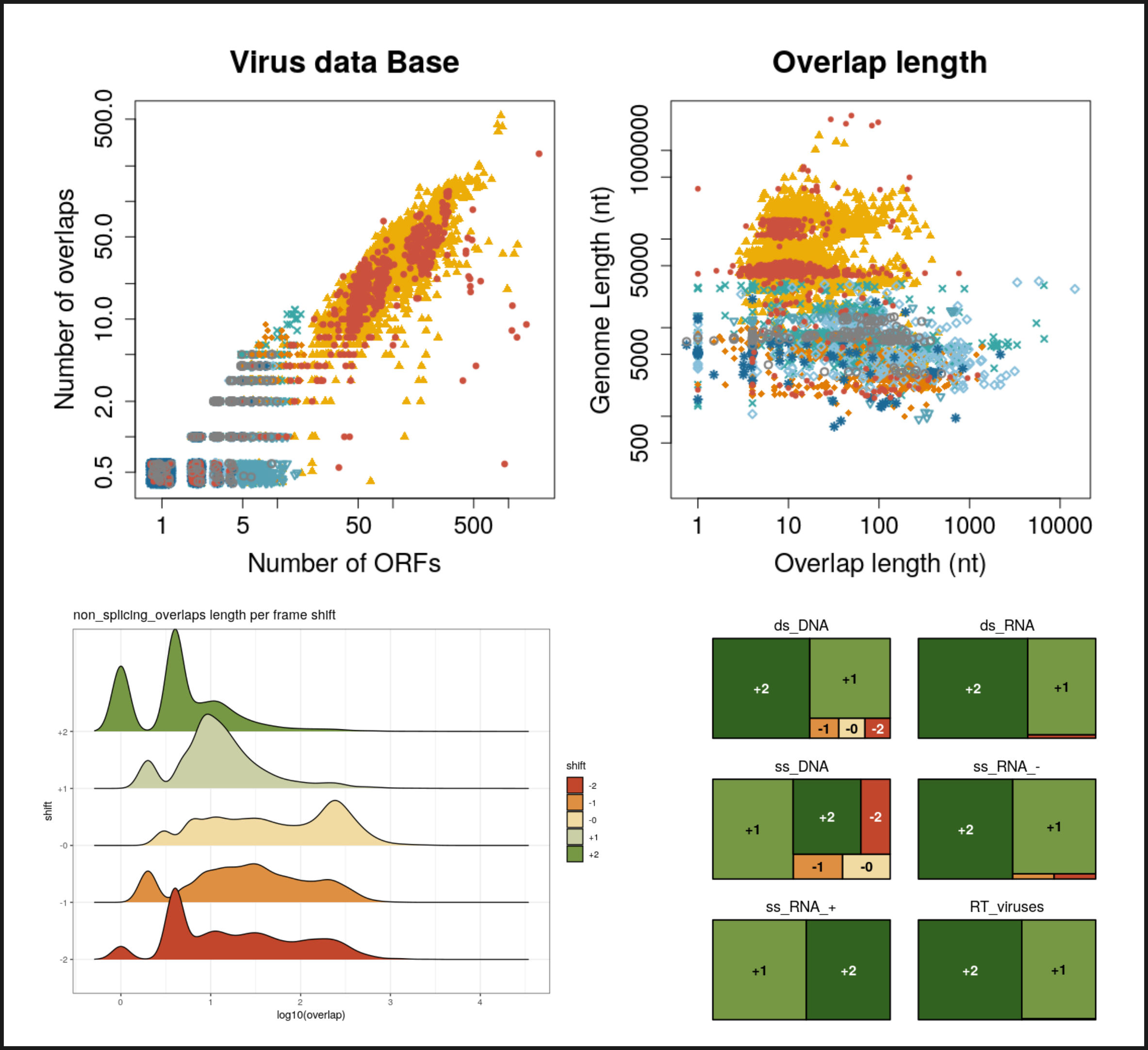

Supplement: S6 Fig — (TIFF) [file ppat.1010331.s006.tiff]

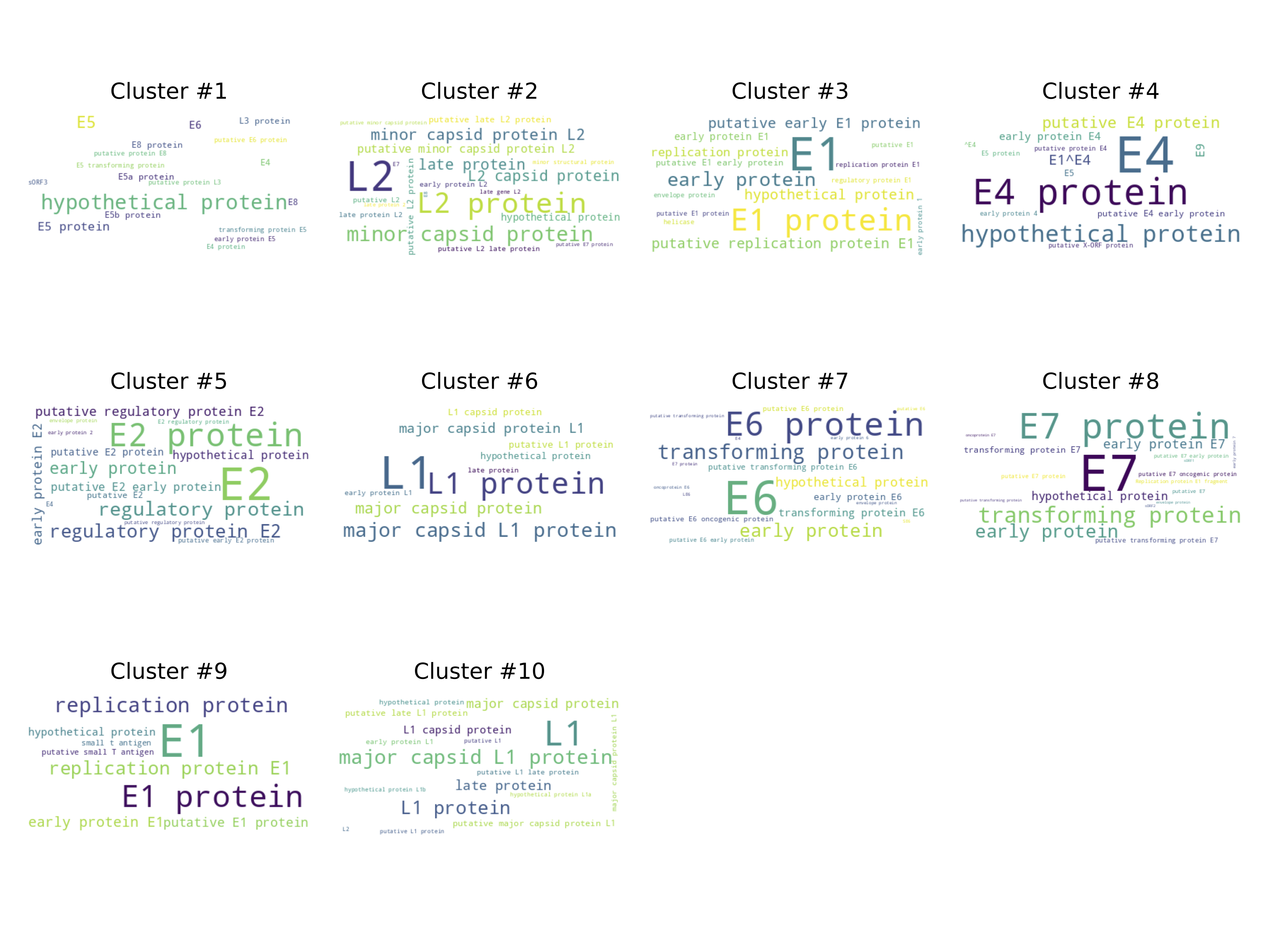

Supplement: S7 Fig — (TIFF) [file ppat.1010331.s007.tiff]

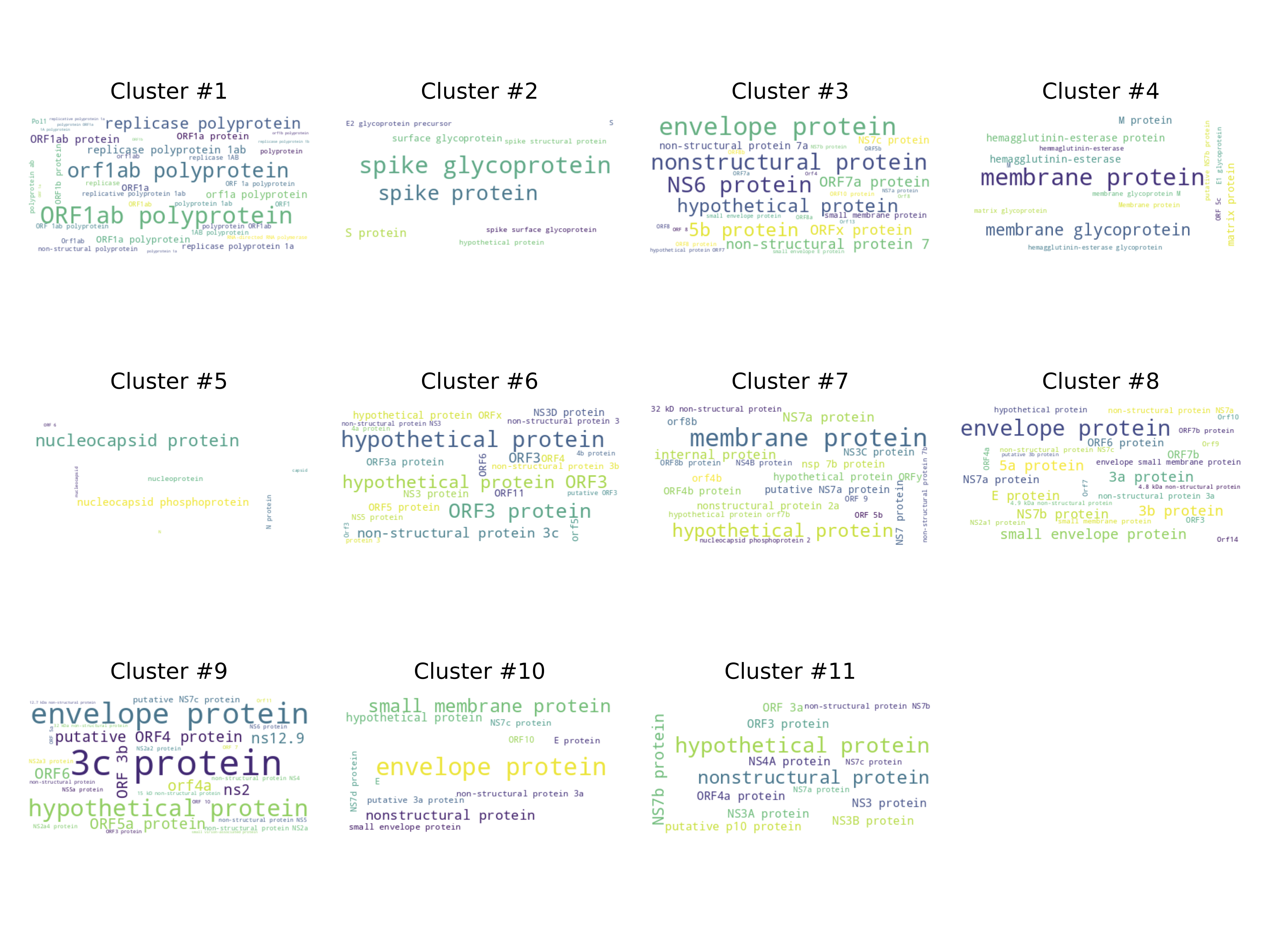

Supplement: S10 Fig — (TIFF) [file ppat.1010331.s010.tiff]
