# Supplementary material for: Using networks to analyze and visualize the distribution of overlapping genes in virus genomes
Source: PLoS Pathog. 2022 Feb 24;18(2):e1010331. doi: 10.1371/journal.ppat.1010331 (PMC8903798; doi:10.1371/journal.ppat.1010331)
Supplement: Attachment — Submitted filename: response.pdf [file ppat.1010331.s012.pdf]

We sincerely thank Dr. Koonin and the two anonymous reviewers for their diligent and insightful review of our manuscript, and for the opportunity to address their feedback with a revised manuscript.

Major Issues

Reviewer 1

> 1. The authors rely on NCBI annotations of CDSs. However, these tend to be quite inaccurate (as the authors discovered for bovine adenovirus NC 002513, p20 lines 5-8). Overlapping and other non-standard genes (ribosomal frameshifting, non-AUG initiation, transcriptional slippage, alternative splicing, misannotation of an upstream in-frame AUG when in fact it is not actually used due to being upstream of a transcript start site, etc) are common in viruses but often dealt with poorly by automatic gene prediction software or indeed by manual annotators. Thus, many virus sequences have missing ORFs, while some sequences may have spurious ORFs annotated as if they were real CDSs.
>   A few misannotations could seriously bias results - particularly for reverse-frame overlapping genes - for example, the authors 4 reverse-frame overlaps in dsRNA viruses arise from "NC_003729" Reoviridae, "NC_042073" Cystoviridae, "NC_043677" Chrysoviridae, "NC_043678" Chrysoviridae and are likely all misannotations.
>   The authors should carefully curate their input dataset and/or show that their methods and conclusions are robust with respect to misannotations.

Thank you for raising this issue. It is a true and unfortunate fact that many records in Genbank are misannotated, even for the reference (RefSeq) genomes used in our study, which were manually curated by the NCBI Viral Genomes Resource initiative. Due to the global scale of our analysis, it was not feasible to manually curate the entire data set of 12,609 genomes (451,228 annotated reading frames). However, there are some quality control measures that can be automated.

For instance, we screened for misannotations by testing whether the length of overlapping regions was consistent with apparent frameshift of the respective reading frames. For example, an overlap between reading frames that are shifted by `+2` should have a length that is not divisible by 3, with remainder 1, *i.e.*, length mod 3 = 1. Applying this filter to our data identified 5,241 (~1%) entries with inconsistent overlap lengths - these entries were

discarded from further analysis. We determined that these cases were caused by undocumented splicing or slippage features. Thus, the cases of misannotation that the reviewer astutely identified had already been filtered out of our analysis, though those entries remained in the source data set they referred to. Due to space constraints, this quality control step was not described in our original manuscript. We sincerely apologize for this misunderstanding, and we have added this explanation to our revised manuscript.

To further evaluate the sensitivity of our results to misannotation, we have performed a simulation experiment in which a random sample of reading frame coordinates were modified. Specifically, we selected a random samples of 10% and 50% of entries in the CSV file containing all coding sequences for viruses in the database, and then shifted the start and end coordinates by an amount drawn from a uniform distribution on the interval -10 to +10 nt (inclusive). We re-ran the analysis pipeline on the modified data to assess the impact of this error variation on our results. In the revised manuscript, we describe this simulation experiment in the Discussion as it relates to database misannotation, and provide the results as Supplementary Material.

> 2. The authors include +0 frame overlaps. By this, they mean where the same coding sequence forms part of two different CDS annotations, e.g. in Venezuelan equine encephalitis alphavirus NC_001449, polyprotein P123 is encoded in the CDS 44-5684 and polyprotein P1234 is encoded in the CDS 44-7526 with readthrough of the stop codon at position 5682-5684. The authors count the region 44-5684 as a +0 frame overlap with the region 44-7526. The authors justification for counting this as an overlap is "Even though the reading frames share codons, they yield different gene products such that the codons are exposed to different selective environments." and "+0 overlaps increase the selective burden of the same nucleotides" (p19 lines 6-10). I find this difficult to swallow. First, the shared domains often have largely similar functions in the two products (e.g. alphavirus P123 versus the P123 domains of P1234) - cf. different-frame overlaps such as orthoreovirus sigma-1 and sigma-1s - where the two proteins have no sequence in common and hence no shared function at all. Second, all codons are subject to multiple selective pressures anyway (translation, mutational, protein function - many proteins are multifunctional); it doesn't make sense to me to single out +0 overlaps as being subject to additional selection pressures.

We concede that sequences belonging to this +0 category of overlaps do not necessarily result in gene products that experience different selective environments. Hence, we have

removed this justification from the manuscript, as well as the corresponding discussion and results from figures and text.

> 3. A second problem with including +0 frame overlaps is that their presence in a genome is very much subject to the whim of the annotator. As an example, the alphavirus NC_001449 has both readthrough and frameshift ORFs annotated:
>   45-5684 and 45-7526 (stop codon readthrough at 5682-5684)
>   7562-11329 and join(7562-9970,9970-10047) (ribosomal frameshift at 9970)
>   leading to $+0$ frame overlaps 45-5684 and 7562-9970. On the other hand, the alphavirus NC_023812 only has two CDSs annotated 43-7467 and 7541..11269. The stop codon read through and frameshift are there, but extra CDSs are not annotated so the authors find zero +0 frame overlaps.
>   Such annotation issues will bias the results as these mainly affect RNA viruses.

We agree that inconsistent annotation of +0 frame overlaps was a significant issue in our original analysis. As noted above, we have removed the +0 overlaps from our main results in the manuscript.

> 4. There seem to be issues with the annotation scripts. For example in the authors' supplementary overlaps.csv file, we have the following entries for NC_001449, listing the +0 frame P123/P1234 overlap in the first line, but then the frameshift ORF join(7562-9970,9970-10047), comprising a +0 frame overlap 7562-9970 and a +2 frame overlap 9970-10047, is listed as two +0 frame overlaps:
>   "NC_001449","non-structural polyprotein precursor P123",44,5684,1,"non-structural polyprotein precursor P1234",44,7526,1,5640,7482,5640,+0 \
>   "NC_001449","truncated polyprotein",7561,10047,1,"structural polyprotein precursor",7561,11329,1,2487,3768,2409,+0 \
>   "NC_001449","truncated polyprotein",7561,10047,1,"structural polyprotein precursor",7561,11329,1,2487,3768,78,+0 \
>   Another example is in simian hemorrhagic fever virus NC_003092, where the 678-nt +1 frame fragment (i.e. 2875-3552) of the join(210-2876,2875-3552) frameshift CDS is labelled as a +0 frame overlap.
>   "NC_003092","ORF1aTF polyprotein",209,3552,1,"ORF1a polyprotein"MaSc,209,6527,1,3345,6318,2667,+0 \
>   "NC_003092","ORF1aTF polyprotein",209,3552,1,"ORF1a polyprotein",209,6527,1,3345,6318,678,+0 \

As described by the reviewer, this issue arises from the presence of internal frameshifts. For example, the record NC_003092 annotates ORFS1ab protein and ORF1aTF polyprotein with coordinates 209:6521; 6520:10996 (indicating a shift of one nucleotide to the right) and 209:2876; 2874:3552 (a shift of two nucleotides to the right), respectively. To calculate overlaps between these ORFs, we treated each portion as a separate fragment, and measured the overlap length on each one independently. As astutely noted by the reviewer, this resulted in two identical entries in the above data file with discordant overlap lengths.

Since such cases are the result of the overlap between two different ORFs, we summed the overlapping lengths on both identical fragments to concatenate the results. In this case, however, the relationship between the frameshifts was not consistent with the length of the overlaps. Thus, these entries and others with the same issue were excluded from our original analysis by our first quality control step, which was not documented in our initial manuscript. We apologize for this misunderstanding, and we revised our manuscript to clarify this quality control procedure and made the post-processing CSV data file available online (https://github.com/PoonLab/ovrf-viz/blob/master/data/filtered_overlaps.csv) so that the data provenance is clearer.

Reviewer #2

> 1. In the graph analysis, homology is based on an alignment free method. how robust are the results of the graph analyses? Will similar conclusions be made with an alignment based homology method? How sensitive are the results and conclusions to the chosen cutoffs? To answer these questions it would be of value to repeat the graph analysis (at least on a small subset) with an alignment based method for homology clustering and possibly also modifying clustering cutoffs on the existing clustering and briefly discuss the impact of these changes on the results and conclusions of the analyses.

This is an insightful point raised by the reviewer. Alignment-free methods, of which many are based on k-mer distances, are generally considered to be more appropriate for the comparative analysis of highly divergent sequences, where large insertions, deletions and genomic rearrangement events may confound conventional methods for sequence alignment. However, it is certainly important to assess the robustness of our results to changing the method for computing distance matrices.

Thus, we investigated the alternative approach of using pairwise alignment to generate a distance matrix for all coding sequences in a virus family. Since alignment is more

computationally complex than matching k-mers, we wrote a message-passing interface (MPI)-enabled Python script to run the alignment program MAFFT in parallel (15 cores) and compute p-distances (*i.e.*, the proportion of aligned sites that are different and ungapped). It was then straightforward to graft the resulting distance matrix into our analysis pipeline. In the revised manuscript, we provide a set of clustering results for Papillomaviridae using pairwise alignment instead of the k-mer-based method as Supplementary Figures, and make a comparison in the Discussion section. In brief, the distribution of gene labels among clusters generated from pairwise alignment distances was not consistent with homology.

> 2. The graph analysis is lacking information of the overlap frame and overlap length, which are of great interest. Adding edges separate for frame and for length in the supplementary information would be of value (or at least adjusting edge width by a minimum of overlap length and discussing the impact on the resulting graph).

We concur that our current layout algorithm for drawing adjacency graphs does not visualize information in the frame or length of overlaps. The key challenge is that there are limited channels for visually communicating information at our disposal, such as node size, edge width, node colour. We have avoided overloading the graph by, for example, varying both node shape, size and colour. As suggested by the reviewer, we revised our Python script for generating graph layouts, and provide an example of scaling edge widths to overlap lengths instead of the number of overlaps as a Supplementary Figure.

Minor Issues

Reviewer 1

> While the authors have referenced previous studies such as Brandes & Linial, Chirico et al, and Schlub & Holmes, I feel that they should also reference Rancurel et al PMID 19640978 and Pavesi et al PMID 30339683, who used carefully curated datasets of overlaps rather than relying on NCBI annotation.

Thank you for bringing these articles to our attention. We have incorporated these references in discussing the trade-off between accuracy and scaleability, with respect to the careful manual curation versus automated computational processing of virus genomes.

> The authors frequently (e.g. p5 line 24, p5 line 25, etc) use the term "reading frame" when the term "open reading frame" (i.e. "ORF") would, I feel, be clearer. A genome has

> 6 (potential) reading frames (viz. +0,+1,+2,-0,-1,-2) but (for large genomes) can have 100s of ORFs.

Thank you for pointing this out. We agree that making the distinction between an "open reading frame" and a "reading frame" resolves the ambiguity of whether we are referring to a coding sequence or a context. We have replaced cases of the former with "ORF" (after first usage of "open reading frame"), and retain "reading frame" for the latter throughout the revised manuscript.

> On p6 lines 10-11, I feel it would be useful to state explicitly how (-)strand ORFs (where the biological 5' and 3' ends are in the opposite orientation with respect to the genome coordinates) are dealt with.

Thank you for your suggestion, we have added a brief explanation to the text: "When genes are encoded on the complementary strand their coordinates keep an ascendant order, and the ORF is tagged with a -1 to indicate the difference in orientation so they can be processed using the same procedure."

> In Fig 4, why are there only 11 clusters in the coronaviridae graph, given that SARS alone has 13 ORFs and there are additional coronavirus accessory genes present in other coronaviruses?

Thank you for raising this point. This is a good example of one of the limitations of clustering ORFs by sequence homology. Our clustering criterion attempts to strike a balance between the generality of a cluster (having at least one representative in every genome) and uniqueness (having no more than one representative in a given genome). This criterion does not readily accommodate uncommonly high biological variation in gene number and identity. For instance, one or more genomes may contain multiple proteins with a high degree of similarity (*e.g.*, from gene duplication in dsDNA viruses), which can become grouped into a single cluster. Conversely, a minority of genomes may contain a unique ORF (through gene gain) that is not sufficient to form a distinct cluster.

In the case of Coronaviridae, only about six ORFs are found across all genomes, with a variable number of accessory genes (about 2 to 8) of low homology. Our adjacency graph for this virus family is consistent with this distinction between conserved and accessory ORFs, with respect to node size, edge weights and distribution of labels. For instance, the graph contains a defined "backbone" of clusters corresponding to the ORFs 1a and 1b, spike (S), envelope (E), membrane (M), nucleocapsid (N). We have incorporated this discussion into the revised manuscript.

> The authors say that simian hemorrhagic fever virus genome (NC 003092) "encodes 33 reading frames" (p9 line 12). I don't know where this number came from because NC_003092 only has ~15 CDSs.

We sincerely apologize for this mistake. The reviewer is absolutely correct - there are only 15 annotated coding sequences in this Genbank record. Our CSV file of ORFs contains the same number, and we recorded 13 overlaps, of which 5 involved `+0` frameshifts that we now discard from our revised analysis. The remaining 8 overlaps involved 10 ORFs. We have corrected this line accordingly.

Reviewer 2

> There seems to be a discrepancy between figure 1c and figure 2a - in overlap of frame +2 the majority of overlap lengths is 1 and 4, and overlap of +2 is the dominant in both dsDNA and dsRNA, however in Figure 1c there are no peaks for dsDNA with length 1 and 4, this does not make sense because there are more dsDNA than dsRNA in the analysis. This apparent discrepancy needs to be fixed or well explained in the text.

We apologize for causing confusion. Figure 1 is an analysis performed at the level of virus genomes, whereas Figure 2 is an analysis at the level of individual overlapping open reading frames (OvRFs). The apparent discrepancy described by the reviewer was the result of overlaps of a single nucleotide being enormously abundant in dsDNA genomes. For each dsDNA genome, this resulted in a single observed mean length close to 1 (Figure 1C), but many data points in the ridgeplot for frameshift +2 (Figure 2, left). We have added an explanation to the text, and corrected the x-axis label for Figure 2, which was erroneously labelled "Mean overlap length" instead of "Overlap length".

> Long overlaps with -0 frame are not explained - is this unique to a single edge between homology clusters or does this occur in several edges, if the latter is true this might have implications for the evolution of the genetic code.

We agree that this is an interesting pattern in the data. In our revised Discussion section, we refer to a recent observation by Lèbre and Gascuel (2017, J Theor Biol) that any combination of residues can be encoded without necessarily inducing a stop codon in the `-0` reading frame, due to redundancy in the genetic code.

> Although most adjacent nodes don't show overlap, some large nodes have thick edges (e.g., nodes 6 and 7 in the Adenoviridae family) suggesting that in some cases

> adjacency of specific genes is accompanied by conserved overlap between the two genes - this should be discussed.

Thank you for raising this point. We have added a discussion of this example in the revised manuscript.
